# Erectile device insertion following phalloplasty in transgender and non-binary individuals assigned female at birth: a narrative review

**DOI:** 10.1038/s41443-023-00764-8

**Published:** 2023-09-22

**Authors:** Giovanni Chiriaco, Aisling Looney, Andrew Nim Christopher, David Ralph, Wai Gin Lee

**Affiliations:** 1 University College London Hospital (UCLH), London, UK; 2 St Peter’s Andrology, London, UK

**Keywords:** Surgery, Quality of life

## Abstract

Genital gender affirmation surgery (gGAS) for individuals assigned female at birth (AFAB) is complex and requires the staged insertion of an erectile device to permit penetrative intercourse. This final stage of gGAS is challenging, owing to the variable anatomy and lack of supportive structures within the neophallus when compared with erectile device insertion for individuals assigned male at birth. There is a paucity in the literature at present regarding erectile device insertion in trans-sex AFAB patients. Hence, a narrative review following a literature review and supplemented by expert opinion from a high-volume centre of expertise is presented. The choices available for erectile device in this patient cohort are discussed. Principle surgical steps required for this complex surgery is outlined along with the recommended postoperative management of the patient. Postoperative outcomes and complications are also summarised in this fast-developing surgical procedure.

## Introduction

The number of individuals assigned female at birth (AFAB) seeking gender affirming therapies for gender incongruence has increased dramatically in recent decades [1], with some studies estimating these individuals account for 0.1 to 2% of the population [2]. The formation of a phallus (phalloplasty) is one of the key surgical steps in genital gender affirmation surgery (gGAS), and primarily utilises autologous tissue transfer to create a neophallus. The aim of phalloplasty is to create an aesthetically acceptable and sensate (tactile and erogenous) phallus that allows both penetrative intercourse and micturition while standing [3].

An erectile device is usually implanted for adequate rigidity during penetrative intercourse. It is expert opinion that surgical insertion of the device should be staged, with a delay of at least 3 to 6 months following the preceding surgery, to reduce the risk of erosion and infection [3, 4]. The insertion of these erectile devices can be challenging due to the lack of the anatomical structures usually present in those assigned male at birth (AMAB) [5]. These procedures should only be undertaken by experienced prosthetic surgeons with an understanding of gGAS.

The first inflatable erectile device inserted within the neophallus for the purpose of gGAS was by Puckett and Montie in 1977 [6]. Earlier devices were prone to higher levels of mechanical failure, particularly in a neophallus [7]. Their durability and resistance to infection have significantly improved over time [8]. However, most erectile devices are designed for individuals AMAB with refractory erectile dysfunction. Cylinders are therefore designed to fit within the corpora cavernosa and the pump to fit discretely within a scrotal sac. The lack of corpora cavernosa in the neophallus requires adjunctive techniques to prevent migration and erosion [9].

This narrative review summarises the contemporary techniques for erectile device insertion in AFAB individuals undergoing gGAS. Outcomes, where available, were also summarised. Discussion will focus on technical details pertinent to this cohort of patients.

## Materials and methods

A Medline and Embase library search of the English language literature was performed to identify the current publications on erectile device insertion in the neophallus of AFAB individuals. Heading terms “transgender persons” OR “gender reassignment procedure” OR “penile prosthesis” OR “gender affirmation” OR “neophallus” were queried, along with similar derivations. Titles, abstracts, and full text articles in the English language literature were screened. Additional studies were identified within the bibliography of screened references.

Only articles pertaining to erectile device insertion in individuals AFAB following phalloplasty were summarised. Emphasis was placed on articles describing techniques in this cohort, but seminal papers that illustrate pertinent technical points were included even if they related to a different patient cohort (e.g. AMAB individuals). Published data were also supplemented by expert opinion from the authors’ cumulative experience in the field.

### Choice of erectile device

There are no comprehensive recommendations to guide surgeons offering erectile device insertion for gGAS despite over 4 decades of practice. The European Society for Sexual Medicine concluded that the current literature was limited by small case series and poor-quality published evidence [10]. Many studies did not include patient reported outcome measures, perhaps due to the lack of validated instruments. Hence, this review summarises evidence amounting to expert opinion given these limitations.

Contemporary erectile devices can be broadly categorised as inflatable devices or malleable rod implants. Inflatable devices have cylinders that can be inflated to desired rigidity using a pump. They traditionally were categorised into either the 3-piece or 2-piece device. Both types consist of one or two cylinders and a pump, but the 2-piece lacks a separate fluid reservoir. The most common prostheses implanted in a neophallus are the 3-piece inflatable devices, namely the Coloplast Titan ^(R)^ (Coloplast, Minneapolis, MN, USA) and the AMS 700 CX™ (Boston Scientific Corp, Minnetonka, MA, USA) [10]. More recently, the ZSI 475 FTM (Zephyr Surgical Implants, Geneva, Switzerland) inflatable erectile device was developed for use in the AFAB population [11]. This device consists of a single cylinder with the distal tip shaped as a glans penis, reservoir, and a pump designed to mimic a testis.

The cylinders of the 3-piece devices are composed of silicone with parylene coating™ (Boston Scientific), Bioflex^(R)^ (Coloplast), 4 layers of silicone and high-tenacity fabric (Rigicon Inc, Ronkonkoma, NY, USA) or 3 layers of silicone (Zephyr Surgical Implants, Geneva, Switzerland). These resemble the rigidity of the engorged corpora cavernosa when inflated. The Boston Scientific, Coloplast and Rigicon devices are not regulated for use in this population but have been adapted for “off-label” use following phalloplasty. The only inflatable device approved for use (CE and UKCA mark) after transgender phalloplasty is the ZSI 475 FTM (Zephyr Surgical Implants). Notably, the Zephyr devices have not been granted regulatory approval in some regions (including the United States). Innovations include a single cylinder device with a removable glans tip and a proximal cylinder plate made of stainless steel and silicone for convenient fixation to the pubic bone [12]. The glans has a ventral indentation that may reduce pressure on the neourethra, and the pump design aims to mimic the appearance of a testis for improved cosmesis. A further recent innovation now has an inflatable testicular prosthesis integrated around the pump unit. The prosthesis can be filled with normal saline via an injection port to resemble a testis more closely.

The AMS Ambicor™ (Boston Scientific) is the only 2-piece device currently available. This fluid-filled device is inflated by releasing fluid from the proximal section of the cylinders using the pump. The cylinders are deflated by bending them for around 10 s. These devices are less easily concealed because of the fluid reservoir within each cylinder and they also lack the InhibiZone™ antibiotic coating found on the AMS 700 3-piece devices.

Malleable rod implants consist of a spiral or solid wire core with a silicone or polyurethane jacket and may be inserted with or without rear tip extenders. The rigidity of the malleable implants remains unchanged whether in the flaccid or erect state, but individuals can adapt the position of the prosthesis as required for sexual activity. They are useful for patients with limited manual dexterity. There are a number of malleable devices available for use in all patient cohorts such as Genesis^(R),^ Coloplast), Tactra™ (Boston Scientific), and Rigi10™ (Rigicon Inc). Zephyr Surgical Implants has also developed the ZSI 100 FTM as a malleable device exclusively for use following gGAS phalloplasty. It has a single adjustable silicone cylinder with glans ‘stopper’ distally and a silicone and stainless-steel proximal plate for easier fixation to the pubic bone. The commonly used devices in this cohort are summarised in Table 1.

**Table 1:** Commonly used erectile devices following phalloplasty in assigned female at birth individuals.

Erectile device type	Product	Product subtypes	Cylinder specifications
Malleable	Boston Scientific Tactra™		Dual-layer silicone
	Coloplast Genesis®		Hydrophilic coating
Two-piece inflatable	Boston Scientific Ambicor™		High-pressure cylinder
Three-piece inflatable	Boston Scientific 700™	CX/LGX/CXR	Parylene Coating™
	Coloplast Titan®	Zero Degree/Narrow Base	Bioflex®
	Rigicon Infla10® X		4 Silicone layers
Phalloplasty-specific	Zephyr ZSI 475 FTM		3 Silicone Layers

### Surgical technique

#### Preoperative considerations

An erectile device is usually inserted in the final stage of gGAS. A neophallus must be constructed first (phalloplasty), using local or distant tissue flaps. Commonly used flaps include the radial forearm free flap, the anterior lateral thigh flap, the abdominal flap and the musculocutaneous latissimus dorsi free flap [13]. Urethral complications should be corrected at least 6 months prior to reduce the risk of infection [4]. Similarly, any ancillary procedures including glansplasty should be completed 6 months prior, to minimise the risk of infection or erosion of the device [13]. Waiting for at least 12 months following phalloplasty may allow sensation to develop in the phallus that may protect from inadvertent trauma [14].

It is important to understand the pertinent anatomical landmarks including the location of the vascular pedicle, clitoris, and urethra to avoid inadvertent injury. This may vary depending on the type of flap used and surgeon or institutional preference [7]. In addition, the phallus should be straight and freely mobile without evidence of contractures. If present, the contractures should be revised to avoid a phallus curvature after surgery.

Erectile device counselling should be offered but this should be informed by surgical factors such as the size and girth of the phallus and size of scrotoplasty. The surgeon will need to assess if one or two cylinders are required. Shared decision making remains paramount, given the prosthetic device options that are now available [15]. Key factors to consider are the individual’s manual dexterity, preferred positioning of the device pump and the type of device they prefer.

All standard preoperative patient factors for penile prosthetic surgery should be optimised [16]. There are no data specific to individuals AFAB following phalloplasty to guide practice so common-sense dictates that these conventions be followed. The following section will focus on the surgical technique to insert 3-piece inflatable devices in the interest of brevity.

#### Preparation and patient positioning

Preparation within the operating theatre should also be consistent with other patient cohorts [16]. Briefly, appropriate intravenous antibiotics based on the local antibiogram should be given. There is no role for anti-fungal prophylaxis despite increasing use in AMAB individuals given all studies to date have not included patients following phalloplasty [17]. Skin should be prepared with chlorhexidine-alcohol skin paint. Consider additional measures to reduce potential microbial inoculation such as limiting traffic within the theatre, placing signage on the theatre door and ensuring all staff are masked despite the lack of strong evidence supporting these practices. Similarly, consider double gloving with changes of gloves prior to handling the device. The patient is positioned supine with their legs parted on a vein board or in dorsal lithotomy to allow easy access to both groin creases if desired [13].

A 14-French indwelling urinary catheter is inserted once the sterile drapes are placed. The neourethra is usually tortuous so a guidewire or catheter introducer may be required. Urethral trauma must be avoided so have a low threshold to use cystoscopic guidance if there are difficulties. Alternatively, a suprapubic catheter can be placed [17] or a catheter omitted as in some centres [18].

#### Choice of surgical incision (Fig. 1)

The primary aim of the incision is to gain access to the pubic body, inferior to the pubic crest. This will be the point of fixation for the erectile device. The incision is further influenced by the location of the vascular pedicle and surgeon preference. A para-scrotal groin crease approach [4], infrapubic approach [13, 19] or perineal approach [20] have all been described.

**Fig. 1:**
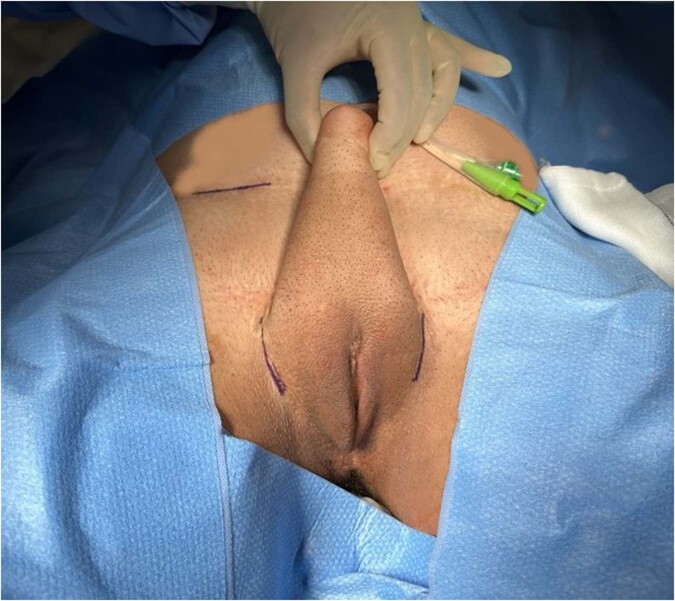
Surgical skin markings for incision. The figure illustrates the surgical marking and incision technique used to gain access to the inferior crest of the body of the pubic bone through a bilateral para-scrotal groin crease incision.

#### Placement of the anchoring sutures (Fig. 2)

Adequate space must be cleared on the body of the pubic bone. Strong non-absorbable sutures such as 1 polyester (Ethibond™) are placed into the periosteum to anchor the proximal portion of the cylinder, or a rear-sheath (“sock”) fashioned from polyethylene terephthalate (Dacron™) or other synthetic material. The number of the anchoring sutures placed is dependent on the number of cylinders implanted. A single cylinder requires at least three evenly spaced sutures, whereas a double cylinder will require at least 6 sutures. Anchoring sutures are typically placed 2 superiorly on the pubis and one inferiorly [4, 20].

**Fig. 2:**
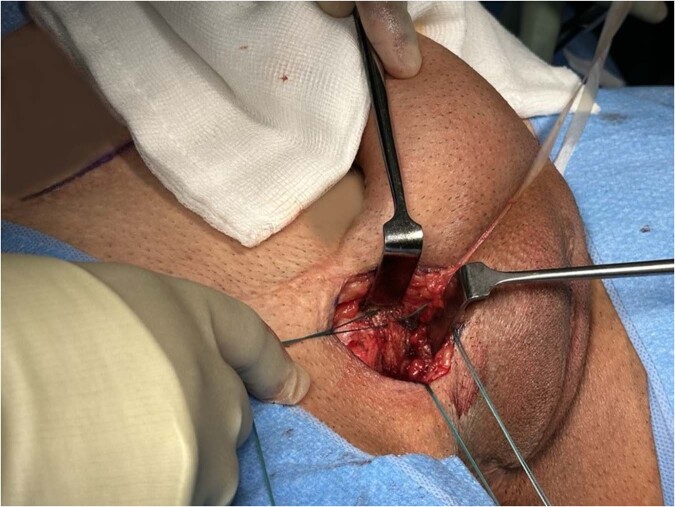
The figure demonstrates the placement of anchoring sutures to ensure stability and secure fixation of the penile prosthesis. Strong non-absorbable sutures, such as 1 polyester (Ethibond™), are anchored into the periosteum on the body of the pubic bone.

#### Neophallus dilatation and cylinder length measurement (Fig. 3)

Space within the phallus is prepared by sequential dilatation with Hegar or Brooks dilators (up to 18 mm if a cap is desired for traditional devices, and up 20 mm if using the ZSI 475 FTM device with glans). An Otis urethrotome can also be considered [18]. The neourethra should be protected during dilatation and a 1 cm cushion of subcutaneous fat between the skin and dilated space should be maintained to minimise the risk of cylinder erosion [7]. The dilated space is then irrigated with an antibiotic solution based on local antimicrobial sensitivities to reduce contamination of the field and to assess for urethral injury. The required cylinder length is measured as the distance between the inferior anchoring suture to the mid glans of the phallus. Cylinder length can vary significantly depending on the neophallus type.

**Fig. 3:**
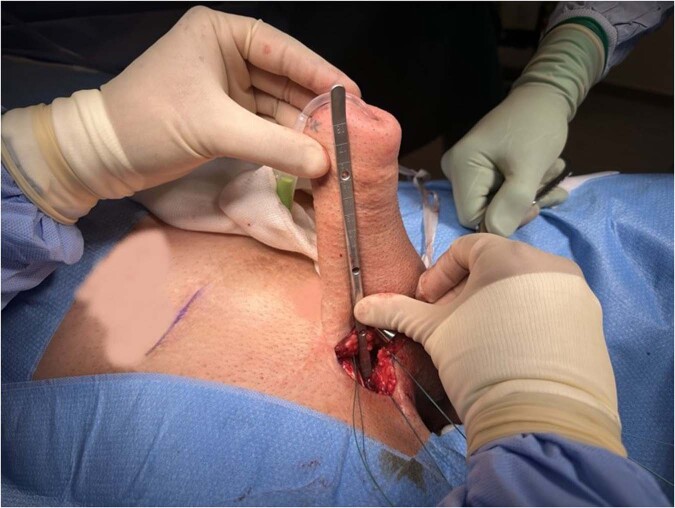
The figure illustrates the process of neophallus dilatation and measurement for the required cylinder length. The measurement of the required cylinder length is performed by determining the distance between the inferior anchoring suture and the mid glans of the neophallus.

#### Reservoir placement

Our preference is to place the reservoir in an extraperitoneal space via a counter incision medial to the anterior superior iliac spine because the anatomical female bladder is usually thinner than those AMAB and more liable to injury. The external oblique aponeurosis is incised and the underlying muscle split so a space in the iliac fossa can be created using a Hegar dilator (18 mm). The reservoir is then placed submuscular while the reservoir tubing is tunnelled under the external oblique aponeurosis or subcutaneously to the base of the phallus to be connected to the pump tubing. A strong running suture in (e.g., 1 polydioxanone) can be used to close the fascia.

Other reported locations for the reservoir include the retropubic space [19], so that it can be reached through the same infrapubic incision used for the anchoring sutures. If the reservoir is placed in the retropubic space, this must be performed contralateral to the flap vascular pedicle. An ectopic submuscular reservoir placement using the same infrapubic incision has also been described [13]. The reservoir volume is filled as per the manufacturer’s recommendations but divided in half if only a single cylinder is used (if the device normally has 2 cylinders).

#### Preparation of the device (Fig. 4)

On a separate sterile trolley, the erectile device is prepared accordingly. A Furlow device or AMS Proximal Tool can be used to measure the developed space to determine the most appropriate cylinder length. If the neophallus will not accommodate both cylinders, one cylinder’s tubing must be divided close to the pump and capped appropriately. For most devices, a polyethylene terephthalate vascular graft (Dacron™) is used to fashion a rear-sheath or sock to cover the proximal end of the cylinder and facilitate anchoring to the pubic bone. A polyethylene terephthalate cap is also fashioned to cover the distal tip of the cylinder to reduce the risk of distal erosion. Both the sock and cap are secured in place with a 2/0 nylon suture. A significant amount of preparation time may be saved if the ZSI FTM devices are used instead due to the inbuilt silicone and stainless-steel proximal fixation plate.

**Fig. 4:**
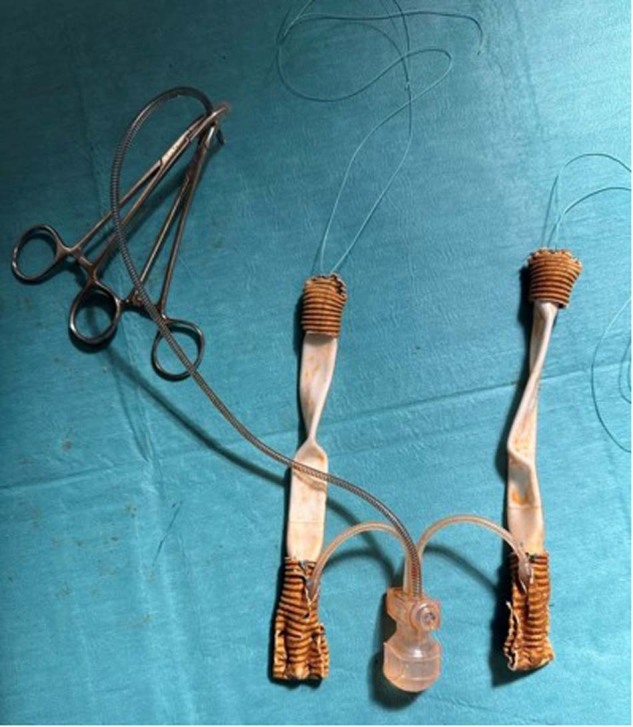
The figure demonstrates the preparation of a 3-piece inflatable penile prosthesis before implantation. A polyethylene terephthalate vascular graft (Dacron™) is utilized to create a rear-sheath or sock, covering the proximal end of the cylinder and facilitating its anchoring to the pubic bone. Additionally, a polyethylene terephthalate cap is fashioned to cover the distal tip of the cylinder, reducing the risk of distal erosion. Both the sock and cap are securely fixed in place using a 2/0 nylon suture.

#### Insertion of the cylinder (Fig. 5)

The cylinder is guided through the previously developed space with the aid of a Furlow and Keith needle. The cylinder requires gentle and considered positioning due to additional friction from the polyethylene terephthalate cap. The polyethylene terephthalate sock is then positioned on the body of the pubic bone and the previously sited Ethibond sutures used to anchor the cylinder. The device is inflated and inspected for appropriate positioning and axial rigidity.

**Fig. 5:**
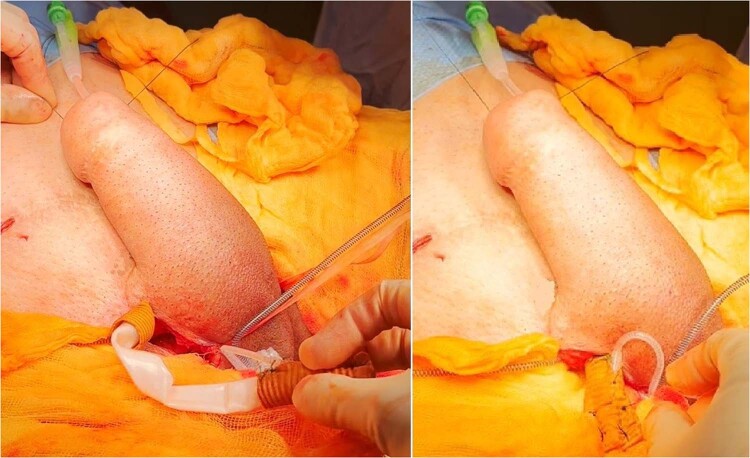
Insertion of the cylinders: the figure illustrates the insertion of the cylinder into the previously developed space within the neophallus. A Furlow and Keith needle are used to guide the cylinder through the space. Once the cylinder is appropriately positioned, the polyethylene terephthalate sock is placed on the body of the pubic bone, utilizing the previously sited Ethibond™ sutures to anchor the cylinder securely.

#### Pump placement (Fig. 6)

The pump is placed within a space that is developed superficially in the ipsilateral neoscrotum or labia majora if scrotoplasty was not performed. The deflate mechanism of the pump should be positioned antero‑laterally for the patient’s convenience. Tubing is connected and then buried. Current evidence suggests that a surgical drain may reduce scrotal haematoma risk following the insertion of a penile prosthesis in individuals AMAB with no increased risk of infection [21]. This debate was recently summarised in an invited commentary [22]. Therefore a 10‑French suction drain is inserted and secured prior to closure. All wounds should be closed in multiple layers to reduce the risk of device erosion or infection (Fig. 7).

**Fig. 6:**
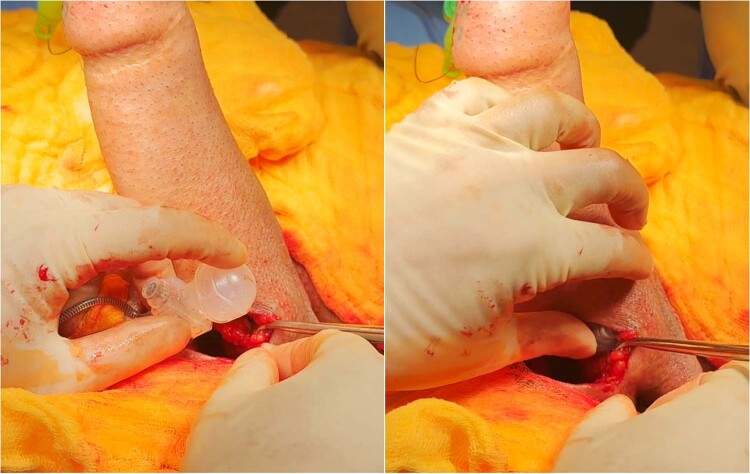
The figure depicts the placement of the pump within a superficially developed space in the ipsilateral neoscrotum. The pump’s deflate mechanism is positioned antero-laterally for the patient’s convenience.

**Fig. 7:**
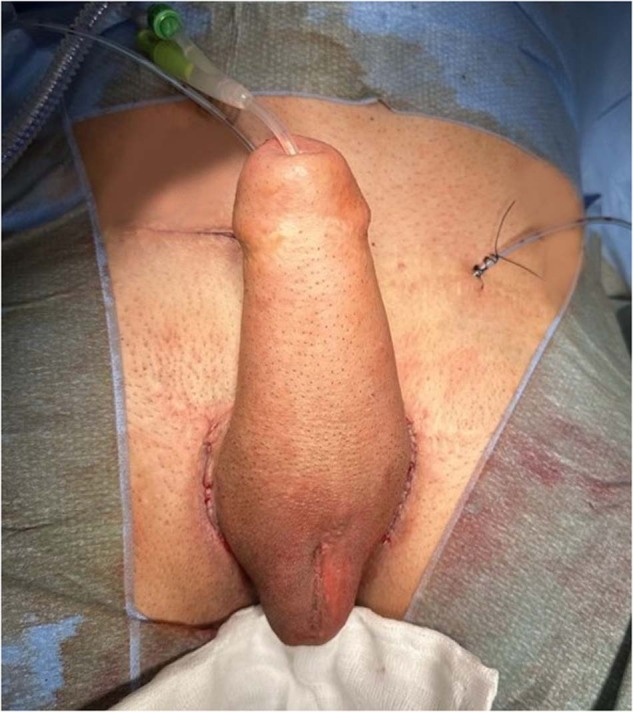
The figure presents the final appearance of the surgical site after the penile prosthesis implantation procedure. A 10-French suction drain is inserted and securely fixed in place to minimize the risk of scrotal hematoma. The surgical wounds are closed in multiple layers.

### Postoperative management

Prophylactic dose low-molecular-weight heparin is administered 6 h post-operatively. AFAB individuals are at higher risk for venous thromboembolism because gender affirmation hormone therapy (testosterone) commonly causes polycythaemia [23]. As described, the surgical technique for inserting an erectile device in these individuals is significantly more complicated leading to longer surgical times (always greater than an hour). They may also be positioned in lithotomy for surgery. Therefore, prophylaxis with low-molecular-weight heparin should be strongly considered in all patients given it does not increase the risk of bleeding [24], unless future studies dispute this. The patient is also prescribed a 5-day course of oral co-amoxiclav, or other suitable agent, depending on allergy status and the local antibiogram. There are no guidelines to direct the most appropriate duration of antibiotic prophylaxis.

The drain can be removed once less than 50 ml has drained within a 24-hour period. Patients undergo a removal of catheter on the first postoperative day and are discharged at this point, with the implant partially inflated for two weeks at approximately 50–60% cylinder capacity. This allows a capsule to form while maintaining appropriate positioning of the distal cylinder tip within the phallus [25]. Inflated cylinders may also reduce the risk of haematoma formation and avoid potential contracture of the tissues post-operatively [4]. Patients are encouraged to begin cycling the device as soon as it is comfortable to do so, usually 2 weeks post-operatively. This should be maintained on a regular basis indefinitely. Penetrative intercourse is permitted from 6 weeks post-surgery.

### Outcomes and complications

Outcomes of erectile device insertion are poorer in this cohort compared to AMAB individuals. The tunica albuginea of the corpora cavernosa measures up to 2.2 mm in thickness (based on cadaveric studies) and is thought to protect prosthetic devices from migration and erosion [26]. The tunica is absent in AFAB individuals, which may explain the higher rates of mechanical failure. The need for grafting material to anchor the device to the pubic bone may also increase the risk of failure due to increased wear on the components. Lastly, most individuals seeking gGAS are significantly younger then AMAB individuals with refractory erectile dysfunction. They are likely to be more sexually active and vigorous when using the devices leading to higher rates of mechanical failure. Noting this, patients should be counselled that many will require revision surgery at some point in their lifetime.

Rooker et al. [27] published a systematic review of outcomes following erectile device insertion for gGAS. From the selected articles, 1032 patients underwent phalloplasty and 689 received one or more erectile devices (total = 824 devices), most of which were inflatable (83.6%). A single cylinder was used in 61% of cases. Mean follow up was 3 years. Most patients (84%) could achieve penetration. Complications, defined as the need for revision surgery, were 45.2% in patients following inflatable penile implantation and 41.5% of those who underwent a malleable device implantation. The most common complications reported were mechanical failure (12%), infection (8.6%), patient dissatisfaction requiring surgery (6.8%), migration of the prosthesis (5.2%), and erosion (3.4%). The most frequently seen mechanical faults were cylinder rupture, followed by aneurysmal deformity of the cylinder, and rupture of the tubing between the pump and cylinder. Reasons for patient dissatisfaction were not described in the review. In the experience of the authors, anecdotal reasons for dissatisfaction include a floppy glans, inadequate axial rigidity of the phallus and excessive girth of the phallus that precludes sexual penetration.

Falcone et al. [4] reported the outcomes for 247 patients who underwent gGAS with insertion of an erectile device in a single centre retrospective study. This study was included in the previously discussed systematic review. All patients could cycle their device following surgery. Seventy-seven percent reported engaging in sexual intercourse, and 61% achieved orgasm. Overall, 88% of this cohort were satisfied with both the cosmetic and functional outcomes. The study reported reduced survival rates of erectile devices when implant in a neophallus compared to those an anatomical penis. The survival rate for erectile devices in a neophallus was 72–79% at 25 months, decreasing to 51–59% at 50 months follow up. There were also increased rates of device malfunction, erosion and infection. No difference in survival rates was demonstrated between the brand of devices used (AMS 700 CX, AMS 700 CXM/R, Coloplast Titan and AMS Ambicor).

One factor found to influence the risk of device malfunction and the need for revision was the choice of flap used for phalloplasty. Patients following abdominal flap phalloplasty required more frequent revision of their erectile device compared to radial forearm free flap phalloplasty. Postulated reasons included the lack of sensation in the phallus and the more frequent need for double cylinder erectile devices given the girth of the phallus following abdominal flap phalloplasty. Wear from both cylinders and the weight of the phallus may be responsible for more device failures [4].

It is the authors’ opinion that the optimal design characteristics for an erectile device intended for insertion into a neophallus would encompass multiple facets; including robustness to minimise the likelihood of mechanical failure, resistance to infection, ease of cycling, separately packaged (not pre-connected) to enable patient-specific adaptation, a pump that resembles a testis to enhance cosmesis, utilisation of soft and durable tubing due to the scarcity of fat tissue in the newly constructed scrotum to bury the tubing, and a self-anchoring capability to the pubic bone with optimal stability.

## Conclusion

Inserting an erectile device following phalloplasty for gGAS is technically challenging and should be undertaken at high volume centres by experienced prosthetic surgeons. Surgical outcomes are poorer in this patient cohort when compared to individuals AMAB. Patient reported outcomes are poorly assessed but suggest acceptable overall satisfaction following surgery.

This field continues to evolve with the advent of novel devices that may be more suitable for individuals AFAB and innovations in the surgical technique. There is an urgent need for higher quality evidence from studies that compare different devices and techniques. Validated questionnaires need to be developed to standardise reporting of patient reported outcomes. Improving the quality of evidence will be instrumental to guide practice and lead to improved patient outcomes.
